# In-Line UV-Vis Spectroscopy as a Fast-Working Process Analytical Technology (PAT) during Early Phase Product Development Using Hot Melt Extrusion (HME)

**DOI:** 10.3390/pharmaceutics10040166

**Published:** 2018-09-23

**Authors:** Walkiria Schlindwein, Mariana Bezerra, Juan Almeida, Andreas Berghaus, Martin Owen, Gordon Muirhead

**Affiliations:** 1 Leicester School of Pharmacy, De Montfort University, Leicester LE1 9BH, UK; mariana.bezerra@dmu.ac.uk (M.B.); juan.zelayaalmeida@dmu.ac.uk (J.A.); 2 ColVisTec AG, 12489 Berlin, Germany; a.berghaus@colvistec.de; 3 Insight by Design Ltd., Stevenage SG9 9ST, UK; martin.owen.insight@gmail.com; 4 GMPharma Ltd., Stevenage SG2 8SB, UK; gordon.muirhead@gmpharma.co.uk

**Keywords:** hot melt extrusion (HME), Quality by Design, in-line UV-Vis spectroscopy, Process Analytical Technology (PAT), continuous manufacturing, piroxicam, Kollidon^®^ VA64

## Abstract

This paper displays the potential of an in-line PAT system for early phase product development during pharmaceutical continuous manufacturing following a Quality by Design (QbD) framework. Hot melt extrusion (HME) is used as continuous manufacturing process and UV–Vis spectroscopy as an in-line monitoring system. A sequential design of experiments (DoE) (screening, optimisation and verification) was used to gain process understanding for the manufacture of piroxicam (PRX)/Kollidon^®^ VA64 amorphous solid dispersions. The influence of die temperature, screw speed, solid feed rate and PRX concentration on the critical quality attributes (CQAs) absorbance and lightness of color (L*) of the extrudates was investigated using multivariate tools. Statistical analysis results show interaction effects between concentration and temperature on absorbance and L* values. Solid feed rate has a significant effect on absorbance only and screw speed showed least impact on both responses for the screening design. The optimum HME process conditions were confirmed by 4 independent studies to be 20% *w*/*w* of PRX, temperature 140 °C, screw speed 200 rpm and feed rate 6 g/min. The in-line UV-Vis system was used to assess the solubility of PRX in Kollidon^®^ VA64 by measuring absorbance and L* values from 230 to 700 nm. Oversaturation was observed for PRX concentrations higher than 20% *w*/*w*. Oversaturation can be readily identified as it causes scattering in the visible range. This is observed by a shift of the baseline in the visible part of the spectrum. Extrudate samples were analyzed for degradation using off-line High-Performance Liquid Chromatography (HPLC) standard methods. Results from off-line experiments using differential scanning calorimetry (DSC), and X-ray diffraction (XRD) are also presented.

## 1. Introduction

The research presented here adopts and evaluates the sequential experimental design methodology using in-line monitoring system based on UV-Vis spectroscopy for early phase product development. In-line sampling is the method of choice for FDA-conforming Process Analytical Technology (PAT) standards. Sampling takes place directly in the process stream and the measurement is performed in real-time. So far, optical spectroscopy has shown to be a suitable in-line technique for hot melt extrusion (HME). The techniques allow non-invasive sampling without interrupting continuous processing [1].

Pharmaceutical HME as a continuous manufacturing (CM) system deserves considerable attention. In the last 10 years, nearly 400 research papers or reviews have been published on CM of pharmaceutical hot melt extrusion (HME) processes, compared to only 60 between 1997 and 2007. Acceptance of continuous manufacturing in the regulatory world helps to pave the way for CM [2]. In July 2015, the U.S. Food and Drug Administration (FDA) approved the use of CM in Vertex’s new cystic fibrosis drug Orkambi^®^ (lumacaftor, ivacaftor) [3]. In April 2016, Janssen Supply Chain (JSC), a division of Johnson & Johnson (J&J), received the first approval to switch from batch to continuous processing for the HIV-1 treatment Prezista^®^ (darunavir) [4].

Pharmaceutical HME process can improve the bioavailability of poorly water-soluble drugs by the formation of solid solutions or solid dispersions [5,6,7]. Recently, AstraZeneca commercialized HME-based tablets Lynparza^®^ (olaparib) for breast and ovarian cancer [8]. It allows embedding of active pharmaceutical ingredients (API) in polymer matrices under operating parameters that can be easily adapted to serve a particular application.

Development phase works towards understanding the parameters that influence the quality of the products. The advantages of a better knowledge of the underlying processes are a higher predictability of the outcome, meaning a lower risk of failure and an improved quality assurance.

The International Conference on Harmonization, ICH Q8 [9] defines this as the Quality-by-Design (QbD) approach: “A systematic approach to development that begins with predefined objectives and emphasizes product and process understanding and process control, based on sound science and quality risk management.” In practice, a QbD approach in HME means implementing predictive and/or statistical models that describe the molecular interactions of API and polymers, their miscibility and the API solubility in the polymer, as well as their physical and chemical stability.

During pharmaceutical HME, API solubility, crystallinity, stability and content uniformity in the polymer mixture are examples of the potentially critical quality attributes (CQAs) that might need to be monitored and controlled [10] 

Near infrared (NIR), mid-range infrared (MIR) and Raman spectroscopy are commonly used PAT systems. Most studies and many reviews published deal with NIR and Raman spectroscopy [11,12,13,14,15,16,17]. However, apart from being costly, the techniques are demanding considering their performance and interpretation. Moreover, they usually lack sensitivity. Meaningful results depend on how well the process is understood and the use of multivariate data analysis tools. Because of the complex setup and interpretation, it may be quite time-consuming to gain reproducible results. 

In contrast, UV-Vis tends to be simple and fast both to set up and to interpret. The short integration time in the millisecond-range delivers rapid results with high sensitivity. UV-Vis therefore is an interesting tool for optimizing an extrusion process. Real-time monitoring with fast results makes it easy to identify and change parameters immediately, thus saving time in testing the critical quality parameters and reproducibility of the results. 

Conveniently, many API show a pronounced absorption in the UV spectrum, between 230–380 nm. Many exclusively or additionally show absorption in the visible part of the spectrum, (380–780 nm) so this wavelength range should also be considered for extrudate characterization, which is shown in this study. Although the chemical information delivered is less specific than NIR and Raman, UV-Vis holds high potential for early phase product development and monitoring manufacturing processes. However, only few studies have been published on the matter [18,19,20], quite often investigating dissolution processes in combination with vibrational spectroscopy [21,22,23,24,25]. 

In this work, UV-Vis spectroscopy alone is used as in-line monitoring system for early phase product development to look at the formation of amorphous solid dispersions of API-polymer as intermediate product to the final tablet formulation. Differential scanning calorimetry (DSC), X-ray diffraction (XRD) and High-Performance Liquid Chromatography (HPLC) are used as off-line characterisation techniques. A qualitative and quantitative evaluation of the extrudates allow rapid statements on API-polymer: color, appearance and solubility threshold limit or oversaturation conditions. A simple way to evaluate these parameters is to measure the changes in absorbance, e.g. at a fixed wavelength, and to calculate lightness (L*) from the spectra. 

The results presented here focus at early development phase. The systematic approach started from identifying a quality target product profile (QTPP) followed by defining critical process parameters (CPPs) and critical quality attributes (CQAs) using a risk-based analysis. Design of experiments (DoE) was based on the outcomes of the risk analysis and results and meaningful conclusions could be reached in no more than one week of experimentation. The aim of this paper is to show the potential of in-line UV-Vis monitoring as a system in continuous processing for fast early phase product development in a very simple, quick and responsive way. The selection of the model system based on PRX was ideal due to its poor solubility and its absorption characteristics in the UV and visible ranges. 

The objectives of this study are to use: in-line UV-Vis spectroscopy as a fast-working PAT tool for early phase product development during HME; a systematic approach based on sequential design of experiments to enhance process understanding, optimize and verify process conditions; multivariate statistical analysis to quantify the relationship between CPPs and CQAs of the extrudates; propose a design space for the process and evaluate the extrudates using off-line techniques to confirm observations made with UV-Vis spectroscopy. Real time data of lightness of color (L*) and absorbance on the visible region were used to identify solubility threshold limit. Oversaturation can be readily identified as it causes scattering in the visible region and a sharp change on the absorbance and L* values is observed. Off-line characterization (HPLC, DSC and XRD) was used to support and validate the in-line measurements.

## 2. Materials and Methods 

**Figure 1:**
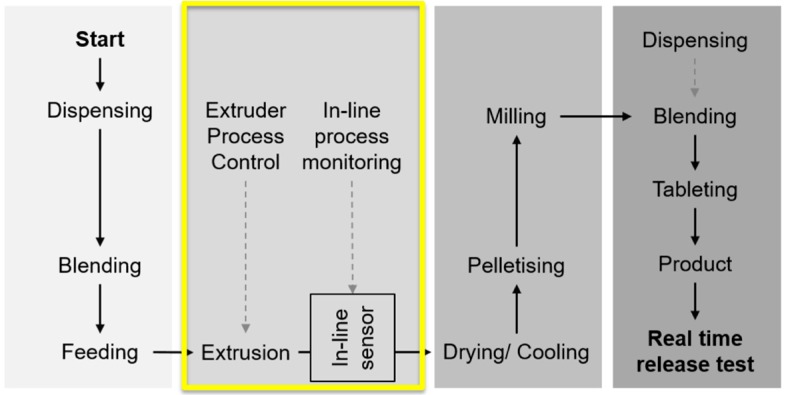
Schematic diagram showing the manufacturing process including the main unit operations from powders to final product, tablets [10]. The focus of this study is on the in-line UV-Vis monitoring of the hot melt extrusion (HME) process. Reproduced with permission from Lundsberg et al., Pharmaceutical Quality by Design: A Practical Approach; published by Wiley, 2018.

A schematic diagram of the pharmaceutical manufacturing process (from powders to tablets) used in this study is shown in Figure 1. For the extrusion experiments, API piroxicam (Medex Ltd., Rugby, UK), a poorly water-soluble, non-steroidal anti-inflammatory drug (NSAID), and poly(vinylpyrrolidone-vinylacetate) copolymer (Kollidon^®^ VA64, BASF, Ludwigshafen, Germany) were used as a model system. The selection of the polymer was based on its physical stability (viscosity, miscibility, solubility) and interactions with the API (dipole intermolecular forces, hydrogen bonding). Kollidon^®^ VA64 has a glass transition temperature close to 100 °C and degradation temperature close to 230 °C. The nature of Kollidon^®^ VA 64 as a matrix forming polymer in HME has been previously studied by researchers to some extent [26].

The main physicochemical properties of Piroxicam (PRX) include [27]: white crystalline solid; four known crystal forms: anhydrate (AH) I, II and III and monohydrate (MH); solubility in water at 22 °C: 23 mg/L; melting temperature (*T*_m_): 198–200 °C; glass transition temperature (*T*_g_): 61 °C; crystallization temperature (*T*_cr_): 60 °C and 83 °C; relative molar mass: 331.35; logP: 3.1 (P = partition coefficient).

The proposed QTPP is for an immediate release solid dosage form (tablet) with a dose of 20 mg of PRX for oral administration [10]. Batches of 250 g API/polymer mixture were blended for 10 min using a tubular mixer. The powder mixture was fed into the extruder using a single screw hopper (Brabender FlexWall^®^, Duisburg, Germany). The solid feed rate was varied from 4–8 g/min (screening design, DoE1).

### 2.1. Hot Melt Extrusion Experiments

**Figure 2:**
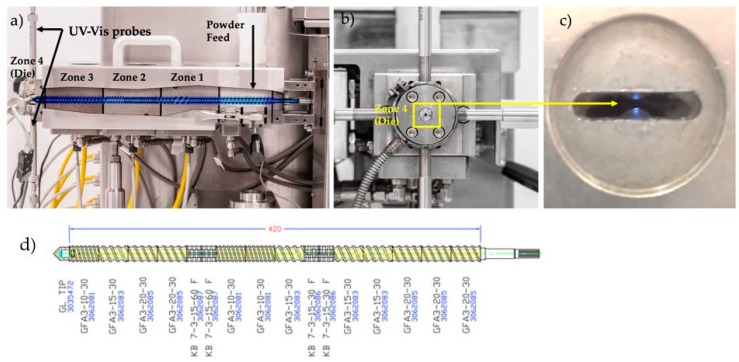
(**a**) Side view of the extruder; (**b**) Front view (zone 4 is where the UV-Vis probes are located), (**c**) View of the probes gap (0.5–0.8 mm) inside the zone 4; (**d**) standard screw configuration set (Leistritz Nano16 with co-rotating twin screws).

Figure 2 shows the extruder Leistritz Nano16 (Somerville, NJ, USA) with co-rotating twin screws. The barrel temperature zones 1 to 3 were set to 120 °C, 130 °C and 140 °C, respectively. The powder feed zone was kept at room temperature. The die temperature, zone 4, was one of the critical process parameters investigated and the values varied from 130–170 °C (DoEs 1&2). The screw elements were conveying (GF-A3-20-30, GF-A3-15-30 and GF-A3-10-30) and kneading (KB 7-3-15-30 F and KB 7-3-15-60 F). The configuration of the screw shaft is shown in Figure 2d.

### 2.2. Risk Assessment and Design Intent

A preliminary risk assessment was carried out for the HME process using the Ishikawa diagram tool [10]. The die temperature, screw speed and solid feed rate were considered as potentially CPPs. The concentration of PRX in the polymer blend was also considered as formulation critical factor that could affect the CQAs solubility threshold limit or oversaturation and appearance of the extrudate. 

For the screening design (DoE1, fractional factorial), ten samples were produced (including two centre points) using the four factors: temperature (150–170 °C), screw speed (100–300 rpm), solid feed rate (4–8 g/min) and API concentration (15–35% *w*/*w*). 

For the optimization design (DoE2, central composite design), only two factors were considered: temperature (130–150 °C) and API concentration (20–30% *w*/*w*). Eleven samples were produced including three centre points. The prediction profiler results from the statistical model (JMP Pro software, SAS Institute, Cary, NC, USA) [28] were used to determine optimum operational conditions that were then verified in the laboratory (verification studies).

### 2.3. In-Line Measurements—UV-Vis

For the in-line measurement, the UV-Vis spectrophotometer Inspectro X from ColVisTec, Berlin, Germany, was coupled to the extruder die. The measurement was conducted with two probes (TPMP, ColVisTec, Berlin, Germany) in transmission mode, using a range of 230 to 700 nm and a sampling rate of 0.5 Hz. The UV-Vis probe has a sapphire window (self-cleaning by material flow) with separable fibre optics for easy calibration. Broad band Xenon flash lamp are used for illumination with fibre length of 5 m. The calibration of the spectrophotometer was performed using the pure polymer, Kollidon^®^ VA64 as a blank reference.

### 2.4. Off-Line Measurements—Differential Scanning Calorimetry (DSC), X-ray Diffraction (XRD) and High-Performance Liquid Chromatography (HPLC)

Thermal analysis, differential scanning calorimetry (DSC) was performed using Perkin-Elmer Jade (Shelton, Connecticut, USA) under N_2_ atmosphere and heating rate of 20 °C/min. Thermal effects were evaluated using Pyris software. 

X-ray diffraction (XRD) was performed using the D2 Phaser X-ray diffractometer (Bruker, Karlsruhe, Germany). The diffraction profile was measured from 5–40° using 2 increments of 0.006. The samples were set to rotate 15 times per minute.

High-Performance Liquid Chromatography (HPLC) was performed on Agilent 1100 (Agilent Technologies, Stockport, UK). The system consists of: a quaternary pump model G1311A, a degasser model G1379A, a column oven model G1316A, a photodiode array detector model G1315B and an autosampler model G1313A. The software Agilent ChemStation Plus (2002) (Agilent, Frankfurt, Germany) was used to control the system and collect data. Chromatographic separations were carried out using a C18 column, Kinetex (250 mm × 4.6 mm, 5 μm) manufactured by Phenomenex (California, USA) at 37 °C, using isocratic mobile phase composed by 45% of methanol and 55% of buffer (citric acid and dibasic sodium phosphate) with flow rate of 1.2 mL min^−1^. 

The method was based on British Pharmacopeia (BP) and United States Pharmacopeia (USP) assays for sample preparation and chromatographic conditions [29,30]. The wavelengths adopted for the BP and USP methods are 242 nm and 254 nm respectively. The UV absorption characteristics of piroxicam, however, allow wavelengths in the range 240 to 400 nm to be used [31,32,33]. The detection method applied for the HME samples considered wavelengths between 330 and 360 nm.

## 3. Results and Analysis

### 3.1. Sequential Design of Experiments 

An essential part of early product development is to have a good understanding of the selected manufacture process, in this case HME and to understand potential failure modes. The first part of this work (10 experiments) was to find the acceptable HME process conditions to produce amorphous solid dispersions of PRX/Kollidon^®^ VA64. Acceptance criteria for “pass” or “failure” were based on the measurements of absorbance and L* of the samples. The second study (11 experiments) was used to optimize the settings. Using this sequential approach, it was possible to suggest an optimum operating condition with only 21 experiments. The results are presented below.

#### 3.1.1. Screening Design

Exemplary results shown in Table 1 and Figure 1 clearly indicate that the ranges used for temperature and concentration for the screening design (DoE1) were beyond the “edge of failure” of the acceptable process conditions. A rapid visualization of the products could be done by considering e.g., appearance and color as “pass” or “fail”. The criteria used for “pass” or “fail” were: transparent uniform yellow samples (“pass”) and dark yellowish/brownish/reddish opaque samples suggesting oversaturation or degradation and/or the presence of bubbles (“fail”).

**Figure 3:**
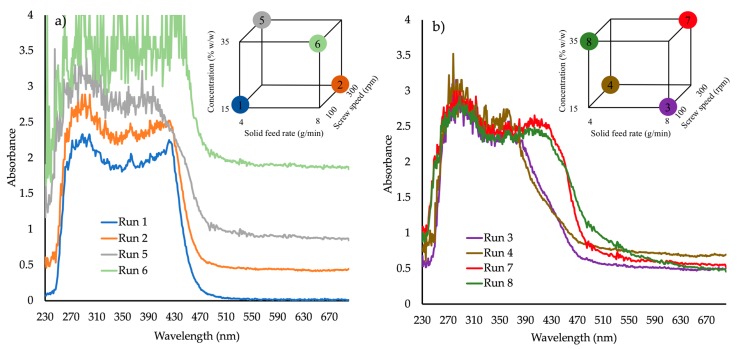
Absorbance vs. wavelength spectra for samples produced at (**a**) 150 °C and (**b**) 170 °C (DoE1, runs 1–8).

Figure 3a absorbance vs. wavelength spectra for samples produced at (a) 150 °C and (b) 170 °C (DoE1, runs 1–8) shows the absorbance spectra of four samples of DoE1 (runs 1, 2, 5 and 6), produced at 150 °C. At this temperature, the only sample that appeared to score “pass” for the selected CQAs was run 1 (15% *w*/*w* API, 100 rpm screw speed and 4 g/min feed rate). It is not surprising that run 6 shows the highest values of absorbance due to its very dark-red color and opaque appearance. 

Figure 3b shows the four samples (3, 4, 7 and 8) produced at 170 °C. It is evident that for high concentrations (35% *w*/*w*) at 170 °C the second absorbance peak is shifted to higher wavelength values (380 to 440 nm) indicating possible different molecular interaction between API/polymer and therefore different phase structures or morphologies. At 170 °C, all extrudates failed. The visible part of the spectra of all “failed” extrudates showed a significant shift to higher absorbance at wavelengths >590 nm.

The in-line UV-Vis instrument measures absorbance and color as a function of wavelength. Numeric color values can be calculated from the spectral transmission measurements across the visible wavelength range 380 to 780 nm; the CIELAB color specification system based on L*a*b*-values describes color differences as seen by the human eye. L* or “lightness” is a color parameter that ranges from black (L* = 0) to diffuse white (L* = 100), b* describes color changes from yellow to blue, and a* from red to green [34]. The statistical analysis of DoE1 was done considering L* and absorbance (at 680 nm) values as responses that represent appearance and solubility threshold limit (oversaturation) as CQAs. The selection of the absorbance at a fixed wavelength was arbitrary, but it was important to select a value in the visible range where low variability was observed (the plateau region), so a simple comparison between samples could be made. 

Statistical analysis of response L* yields a linear model with an F Ratio (signal to noise) of 210.83 (Table 2). The adjusted R^2^ value indicates the model explains 99.76% of the variability. Concentration and screw speed were shown to be statistically significant, as were the concentration-solid feed rate interaction and the concentration-temperature interaction. The main effects for temperature and solid feed rate were included in the model because they are involved in statistically significant interactions with concentration.

Statistical analysis of response Absorbance (Abs) at 680 nm yields a model with an F Ratio (signal to noise) of 1276.3 (Table 3). The adjusted *R*^2^ value indicates the model explains 99.99% of the variability. Note that if only linear terms were included, then the signal to noise ratio drops to less than 5 and the adjusted *R*^2^ value indicates the model explains less than 55% of the variability. The inclusion of two centre points in the design allows curvature of one of the factors to be estimated. By assigning this to the most important main effect (concentration) the model fits are much improved. However, in this design, the concentration quadratic term is aliased with the quadratic terms of the other factors, hence a central composite design (DoE2 Optimization design) was required to resolve this ambiguity of curvature assignment.

**Figure 4:**
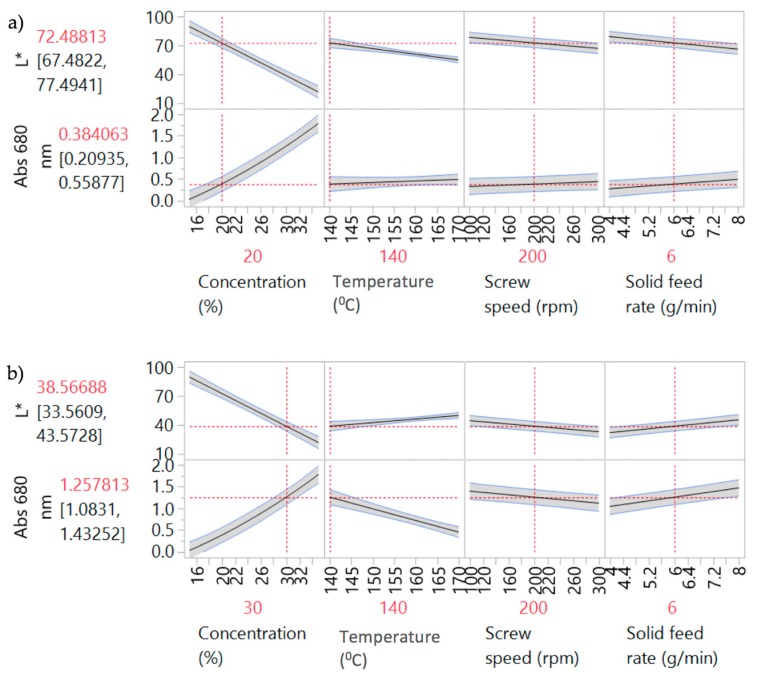
Prediction profilers for DoE1 for (**a**) 20% *w*/*w* and (**b**) 30% *w*/*w* PRX concentrations.

Figure 4 shows the prediction profiler plots (JMP software) [28] for the four factors (screw speed, temperature, solid feed rate and concentration) and two responses (L* and Abs at 680 nm). Concentration, temperature, screw speed and solid feed rate are plotted against the changes of L* and Abs at 680 nm in DoE1 setting. The success criteria for the Abs at 680 nm and L* are to achieve minimum (close to zero e.g., <0.1) and maximum (close to 100 e.g., <90) values, respectively.

Concentration was the predominant critical parameter for both responses. The prediction profilers are shown for different settings of concentration in order to show the impact of the interaction terms. When the concentration is lower (Figure 4a) the temperature has little effect on Abs at 680 nm. When the concentration effect is higher (Figure 4b), temperature has an inverse impact on Abs at 680 nm. Close to saturation, (in this case for concentrations >20% *w*/*w*) temperature becomes an important parameter. Solubility tends to increase with temperature and the responses absorbance at 680 nm and L* are expected to decrease and increase respectively as the temperature increases. Solid feed rate has a significant effect on absorbance at 680 nm only, and screw speed showed least impact on both parameters for these experiments. As discussed earlier, the initial ranges used for the factors studied in DoE1 were defined based on the physical chemical properties of the API and polymer. This initial design (screening) resulted in most samples appearing burnt, i.e., possibly degraded. HPLC results are shown in Section 3.3.3. Consequently, these conditions were beyond the “edge of failure” for the process and not to be used. A new design was proposed to identify the operating space.

#### 3.1.2. Optimization Design

New ranges of temperature and concentration were investigated for the optimization study of the process conditions. Table 4 shows the runs for DoE2, central composite design. 

Statistical analysis of response L* yields a model with an F Ratio (signal to noise) of 61.5 (Table 5). The adjusted *R*^2^ value indicates the model explains 96.03% of the variability. Concentration, temperature and the concentration-temperature interaction were shown to be statistically significant. The concentration quadratic term is also significant.

Statistical analysis of response Abs at 680 nm yields a model with an F Ratio (signal to noise) of 109.6 (Table 6). The adjusted *R*^2^ value indicates the model explains 97.75% of the variability. Concentration, temperature and the concentration-temperature interaction were shown to be statistically significant. The concentration quadratic term is also significant. 

**Figure 5:**
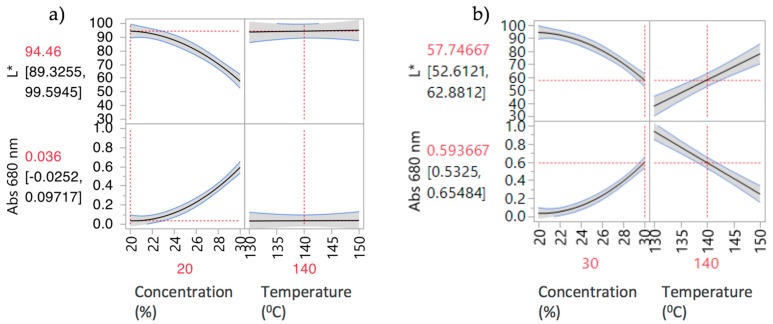
Prediction Profilers for DoE2 (**a**) 20% *w*/*w* and (**b**) 30% *w*/*w* concentrations.

Figure 5 shows the prediction profiler and interaction effects for DoE2. Curvature behaviour was observed for concentration. As expected, there is a strong interaction effect between concentration and temperature. At low concentrations (Figure 5a), temperature has little effect on both responses; i.e., L* and absorbance are insensitive to temperature change at 20% *w*/*w*. The API is completely soluble at this concentration, so increasing the temperature has no effect on both responses. At high concentrations (Figure 5b) there is a strong effect due to temperature. Increasing temperature leads to an increase of L* and decrease of Abs at 680 nm values, reflecting lower solubility at lower temperatures. Samples 4–11 have “failed” the criteria for acceptable process conditions due to mainly solubility issues (oversaturation) or presence of bubbles. The "failed" samples were outside the intended design space and, hence, potential operating space.

Based on the way the spectroscopic system was calibrated, the “lightness” (L*) represents the API concentration if the API is in solution. If it is not fully dissolved, particles of undissolved API will scatter the light out of the transmission path, which reduces the intensity (light vs. dark) [35,36]. This non-selective scattering effect is wavelength independent, as particles are larger than the wavelength of light. The L* value is very sensitive to this effect, making it a very convenient quantitative tool for immediate visualization of oversaturation—without any need of a full spectral analysis.

**Figure 6:**
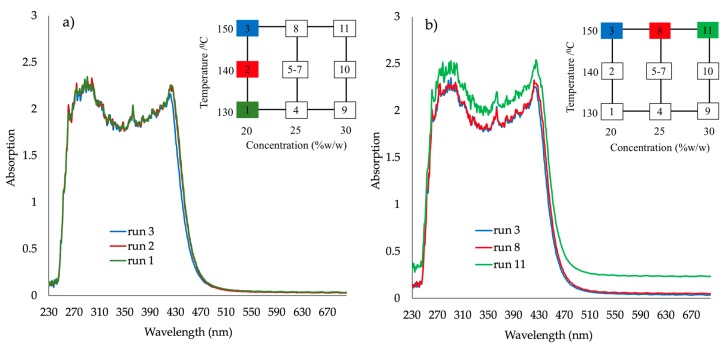
Absorbance spectra for the response surface design runs (DoE2) (**a**) API 20% *w*/*w* at 130 °C (run 1) 140 °C (run 2) and 150 °C (run 3); (**b**) temperature at 150 °C for API concentrations 20% *w/w* (run 3), 25% *w*/*w* (run 8) and 30% *w*/*w* (run 11).

An increase of absorbance intensity (e.g., Abs at 680 nm) could be observed as the concentration changed from 20 to 30% *w*/*w* of PRX at 150 °C (Figure 6b). Oversaturation led to a significant shift of the absorbance values that is also influenced by decreasing temperature. For PRX 20% *w*/*w*; run 1 *T* = 130 °C; run 2, *T* = 140 °C; run 3, *T* = 150 °C; samples 1, 2, 3 are transparent and yellowish, “passed”.

For samples prepared at 150 °C and concentrations 20–30% *w*/*w* (Figure 6b runs 3, 8, 11) the oversaturation with 30% *w*/*w* concentration is clearly shown. Absorbance plateau in the visible spectra range shifts to higher absorbance with increasing API concentration (run 11), indicating higher opacity, i.e., oversaturation.

**Figure 7:**
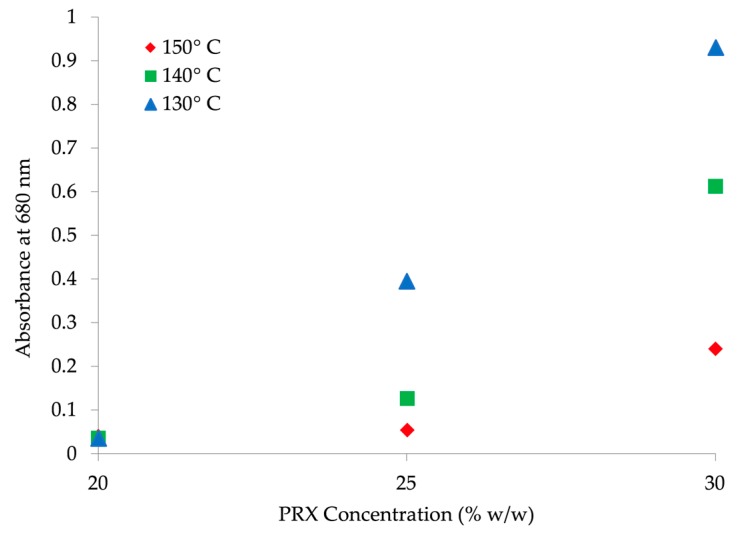
Absorbance at 680 nm vs. API concentration (% *w*/*w*) at 130 °C, 140 °C and 150 °C.

Figure 7 clarifies the relationship between the two factors and API solubility. Absorbance at 680 nm increases with higher concentration and lower temperature, suggesting that solubility increases with higher concentration until the point of oversaturation. At 20% *w*/*w* API, no influence of temperature on solubility is observed. As expected, with increasing API concentration, (25% to 30% *w/w*) and decreasing temperature (150 °C to 130 °C), an increasingly pronounced absorbance shift is observed, indicating oversaturation.

#### 3.1.3. Process Parameters Verification

**Figure 8:**
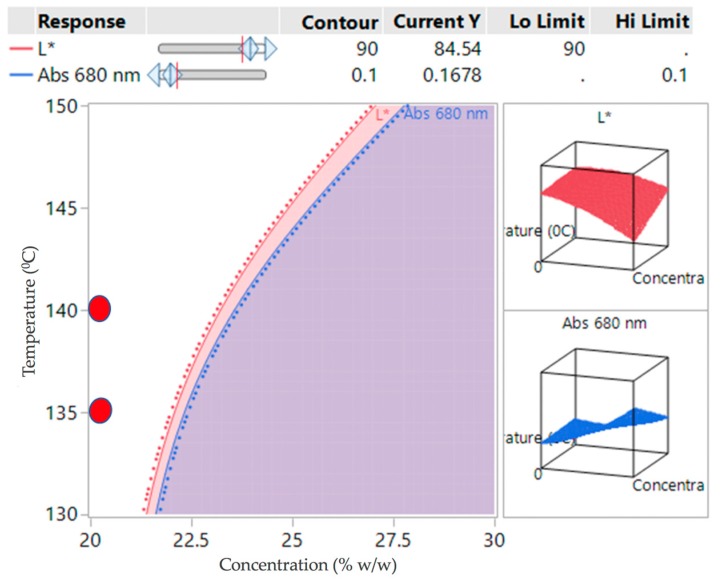
Contour plot for DoE2 indicating a potential design space (white region) overlaid verification study conditions (red circles).

If the criteria for success is set that L* must be greater than 90 and Abs at 680 must be less than 0.1, then the white region in Figure 8 indicates a potential operating space where the samples can be produced. The red circles indicate the operating conditions investigated in the verification study.

The DoE2 study showed that the potential design space (the white region) was at the edge of the investigation space (Figure 8). Lowering the temperature from 170 °C prevented apparent degradation of the samples, however, processability at 130 °C was difficult due to the high viscosity of the polymer, causing high torque. For concentrations at 25% *w/w* or above most samples appeared to be opaque, indicating oversaturation of API. 

For this reason, the verification runs (Table 7) were carried out between 135 and 140 °C at 20% *w*/*w* concentration (small red circles in Figure 8). For further assurance of ruggedness, the verifications were run on different days by different operators at different screw speeds (200–300 rpm), keeping the feed rate constant at 6 g/min and concentration of 20% *w*/*w*. 

**Figure 9:**
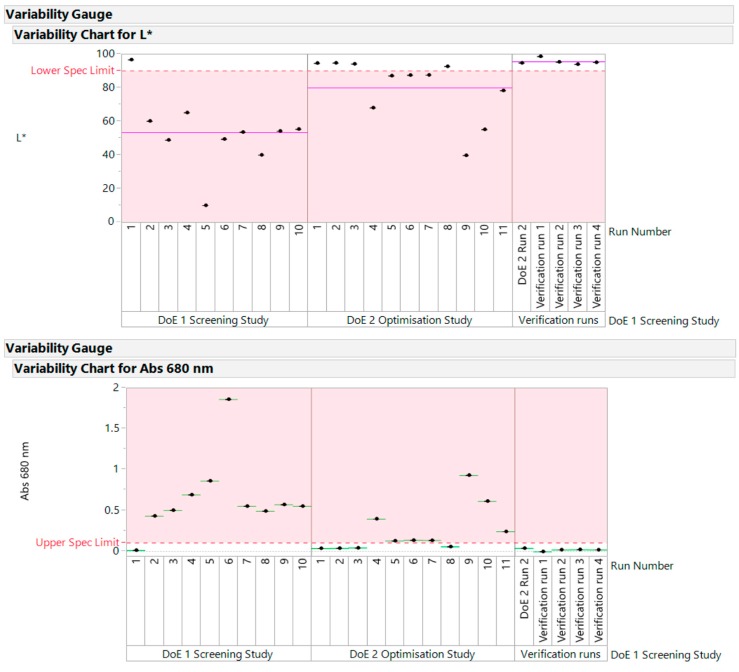
Variability gauge plot for data across all studies.

All the verification runs produced light yellow, transparent samples that were acceptable. They confirmed the conclusions drawn from DoE1 and DoE2. The variability gauge plot for data across all studies is shown in Figure 9. The samples above the lower limit for L* and below the upper limit for Abs at 680 nm “passed” the quality criteria for this early development phase.

### 3.2. In-Line UV-Vis Spectral Visualization

**Figure 10:**
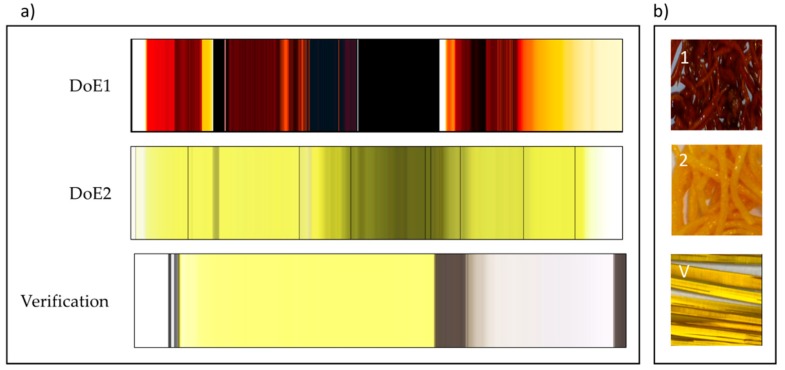
(**a**) Visualization of absorbance spectra of all experiments: DoE1, DoE2, verification. DoE1: ≈2000 spectra collected in 5 h; DoE2: ≈1500 spectra collected in 3 h; Verification: ≈650 spectra collected in 2 h. (**b**) Representation of samples from each DoE showing color and appearance of extrudates: (1) DoE1 opaque and very dark red (run 6: API 35% *w*/*w*, *T* = 150 °C, 100 rpm, 8 g/min); (2) DoE2 opaque and slightly orange (run 10: API 30% *w*/*w*, *T* = 140 °C, 200 rpm, 6 g/min); (V) Verification, transparent light yellow (run: API 20% *w*/*w*, *T* = 140 °C, 200 rpm, 6 g/min). Reproduced with permission from Lundsberg et al., Pharmaceutical Quality by Design: A Practical Approach; published by Wiley, 2018.

The spectra for each DoE set of runs were recorded continuously and are shown as vertical color bands from left to right in Figure 10a. Each row consists of several 100 to 2000 color strips representing the sequence of spectra of each DoE run (10 runs for DoE1 and 11 runs for DoE2). The colors represent the visible part of the spectra, measured in transmission, of the mixture PRX/Kollidon^®^ VA64. Colors as seen in Figure 10 were calculated from the spectra based on transmission measurements and expressed as L*, a*, b* values, thus representing colors as seen by the human eye [34]. Conversion to the RGB color mode adapted the colors to computer screens and for printers. In the last set of spectra, highlighted as verification experiment, the dark-grey color is from the cleaning polymer material (opaque). The white color spectra are pure polymer, without PRX. Rapid changes in color from yellow-grey shows the formation of bubbles within the extruded API/polymer sample. This could be due to the presence of moisture. The color red/orange and black suggests deterioration of API due to excess temperature/concentration (DoE1). The light yellowish color suggests optimum operational condition producing homogenous transparent API/polymer mixture. 

The acceptance ranges for temperature, API concentration, screw speed and solid feed rate were determined with only 21 experiments and 500 g of API in one week. Using a risk-based approach and UV-Vis as a PAT system, it became quickly clear that, for this system, temperature and concentration of API are critical to product CQAs (e.g., solubility threshold limit and L* of the extrudate). 

### 3.3 Off-Line Measurements—DSC, XRD and HPLC 

#### 3.3.1. Differential Scanning Calorimetry

**Figure 11:**
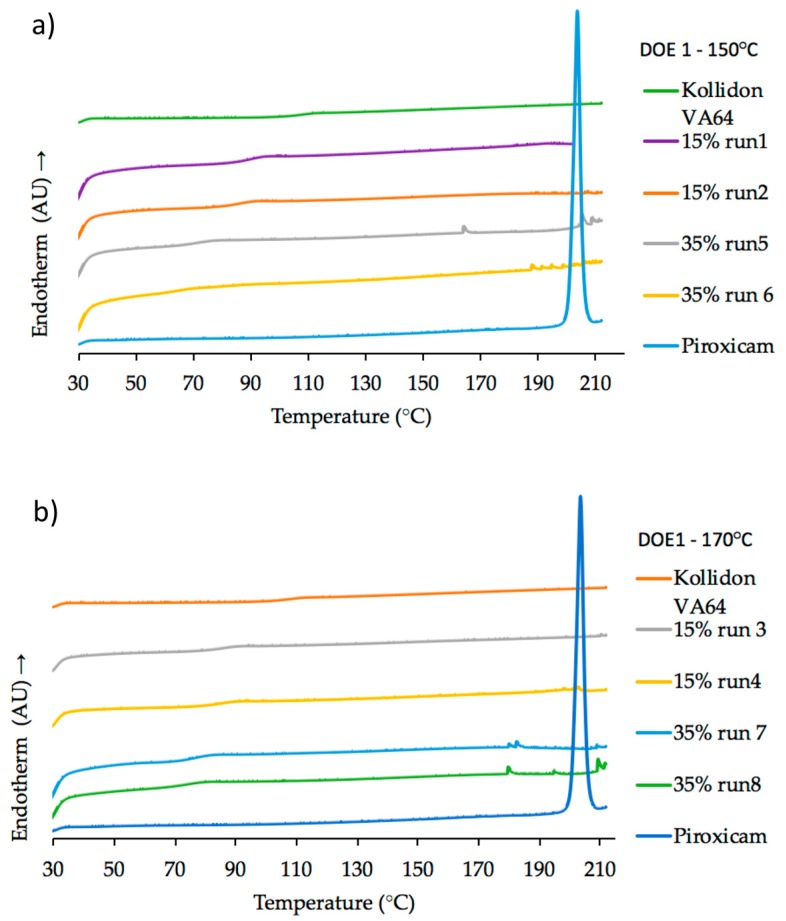
DSC heating curves (20 °C/min) of pure PRX, pure Kollidon^®^ VA64 as reference and samples from (**a**) DoE1 at 150 °C; (**b**) DoE1 at 170 °C.

The polymorphism of PRX has been discussed by many authors [37,38,39] and several analytical techniques (DSC, XRD, FTIR, NIR, Raman, etc.) have been used to characterize the different crystal forms of PRX [40,41,42,43]. DSC result for pure PRX (onset melting temperature, *T*_m_ = 202.7 °C) is in good agreement with the results reported by Vrečer et al. [38,39] as crystal form I. The glass transition temperature (*T*_g_) of pure Kollidon^®^ VA64 (101.5 °C) is also in agreement with values from literature [26]. Exemplary thermograms for DoE1 runs are shown in Figure 11a, b. Glass transition temperature values decreased as the concentration of PRX increased from 15% *w*/*w* (*T*_g_ = 84.8 °C) to 35% *w*/*w* (*T*_g_ = 71.6 °C).

For HME process temperatures equal or higher than 150 °C (DoE1) except Run 1, all samples had a dark orange/red color suggesting possible degradation, as shown in Figure 10. As mentioned before, these conditions were beyond the “edge of failure” for the process. However, the knowledge acquired from these experiments was important to define the next set of experiments (DoE2). Kogermann et al. [43] have reported that the presence of degradation products [44] can act as plasticizers, which support the observed decreasing in *T*_g_ values. There is a strong interaction effect between concentration and temperature, as described earlier (Figure 4). 

Thermograms of samples with PRX concentrations of 15% *w*/*w* (DoE1) and 20% *w*/*w* (DoE2 and verification) confirmed the presence of amorphous PRX in Kollidon^®^ VA64 formulations.

#### 3.3.2. X-ray Diffraction

**Figure 12:**
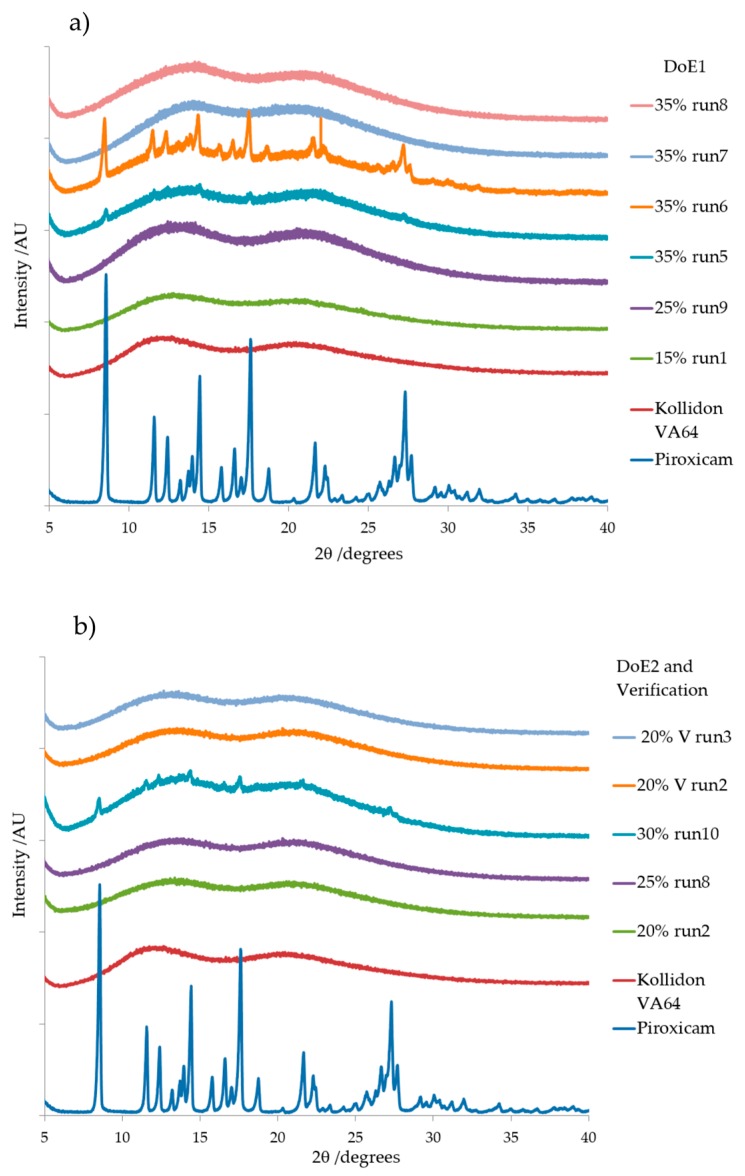
XRD diffraction patterns of (**a**) DoE1 runs and (**b**) DoE2 and verification runs. Pure PRX and pure Kollidon^®^ VA64 are shown as reference. Note: V = verification.

The diffraction pattern (Figure 12a,b) for pure PRX is in good agreement with previously published data [38,39,43] as crystal form I. Kollidon^®^ VA64 XRD pattern is characteristic of an amorphous material with no sharp and intense peaks [45,46]. The XRD patterns for selected runs from DoE1, DoE2 and verification runs are shown in Figure 12a,b. There was no sign of crystallinity or characteristic peaks at specific 2*θ* angles for samples with PRX concentration of 15% *w*/*w*, 20% and 25% *w/w*. This indicated the formation of an amorphous solid dispersion in those formulations. This data further supports the DSC data discussed earlier where the active was completely miscible in the melt extrudates up to 20% *w/*w** in which there was no sign of the characteristic melting peak of PRX. 

Samples with PRX concentrations 30% *w*/*w* and 35% *w*/*w* (e.g., Run 6, DoE1 and Run 10 DoE2 showed to be partially amorphous, exhibiting relatively less intense and more diffused peaks. The 2*θ* angles of the peaks remained unaffected. These changes in peak intensity could be attributed to the solubility of PRX in the Kollidon^®^ VA 64 matrix, and the unchanged 2*θ* angles of the peaks indicate existence of original crystalline form of the drug (form I) as oversaturated crystal phase. In contrast, samples with 35% *w*/*w* of PRX that were processed at 170 °C have shown no XRD patterns. This could be due to either degradation or excess PRX being re-dissolved (DoE1 runs 7 and 8).

#### 3.3.3. High-Performance Liquid Chromatography 

The concentrations of the HME samples (DoE1, DoE2 and verification runs) were calculated using 330 nm and 360 nm as the selected wavelengths. Table 8 shows sample nominal concentration, average area, standard deviation, coefficient of variance, measured concentration and relative areas of Peak A and Peak B. Peak A (pure PRX, retention time 5.3 min) is observed in all standard solutions used for the calibration curve experiments and all HME samples. The smaller peak (Peak B) which elutes at 2.4 min is not present at 360 nm for pure PRX or blank chromatograms.

**Figure 13:**
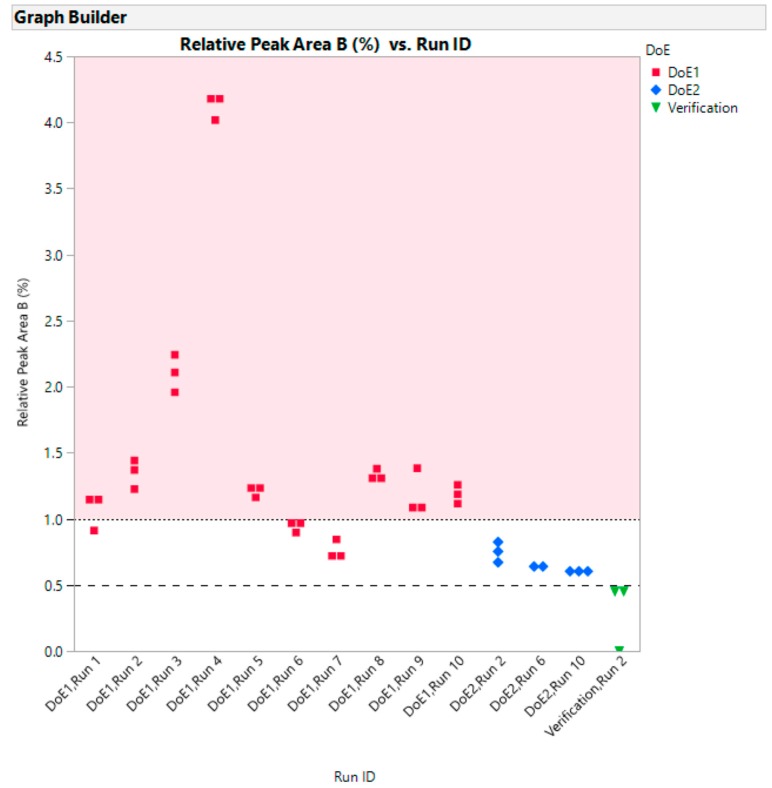
Relative peak area (three injections) Peak B vs Run ID for DoE1, DoE2 and verification runs.

It should be noted that the correlation between the relative percent of Peak B does not correlate particularly well with the appearance of the run samples described earlier in Table 1. This suggests there may be other impurities present which have not been resolved from the main peak. Further work is required to investigate this. However, it is possible to show (Figure 13) that as we progress from the screening design to the optimization design to the verification run that the level of Peak B drops.

**Figure 14:**
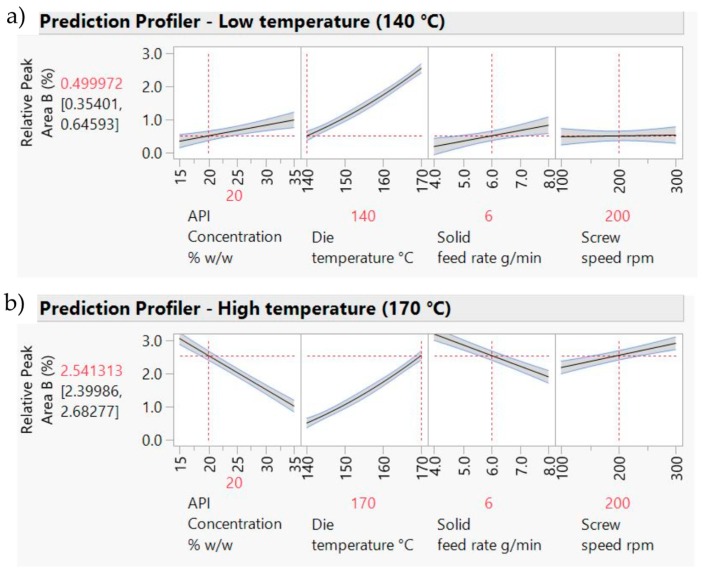
DoE model showing the impact of process parameters on the Relative area of Peak B. (**a**) Prediction profiler for 140 °C; (**b**) Prediction profiler for 170 °C

A DoE model fitted to the HPLC data (Table 9 and Figure 14) indicates a good model explaining ca.94% of the variation. Die temperature has the most important impact on the relative area of Peak B. As temperature increases, the other factors become more important (i.e., there are interaction effects between temperature and the other three factors).

### 3.4. Extrudate Concentration Studies 

**Figure 15:**
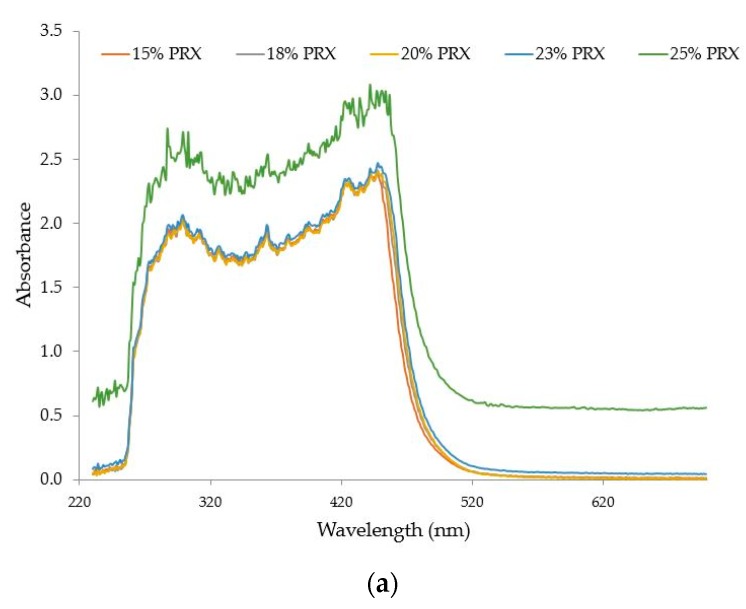

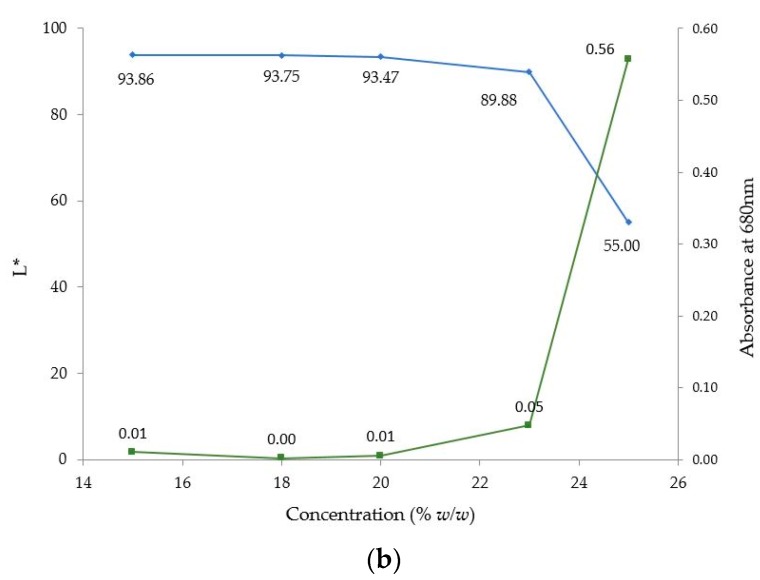
(**a**) Absorbance spectra of extrudate with PRX concentrations from 15% to 25% *w*/*w*. (**b**) L* and absorbance values at 680 nm of PRX/polymer extrudates with API concentrations from 18 to 25% *w*/*w*.

Five samples with PRX concentrations from 15 to 25% *w*/*w* were processed at 140 °C, 200 rpm and 6 g/min. Figure 15a shows the absorbance spectra versus wavelength (nm) for these five samples. The observed shift of the baseline was not due to the polymer but to the excess of API (oversaturation) in samples with concentrations higher than 20% *w*/*w*. Undissolved API causes scattering which is characteristic of a baseline shift in the visible region. Scattering can also be observed if bubbles are present, but this was not the case for the process conditions used during these experiments. The values of absorbance at 680 nm and L* as a function of PRX concentration (15–25% *w*/*w*) are shown in Figure 15b.

The decrease in L* and increase in absorbance values for samples with concentrations above 20% clearly confirm the solubility limit threshold for this system, which is between 20–23% *w*/*w* of PRX.

## 4. Discussion

The results showed that qualitative and quantitative assessment of the state of the extrusion process based on observation of raw spectral data is a very useful system during extrusion. 

A model example was chosen where the quality of the extrudate is visible by color and appearance to the naked eye. Both parameters, color and appearance, already belong to visual pharmaceutical quality standards, and it is common that the human eye serves as an instrument to judge purity or identify contamination during a pharmaceutical manufacturing process. However, these visual methods are highly subjective and do not deliver quantifiable data. An initial investigation of purity by HPLC identified a potential degradant (peak B) as a potential source of the color. A more rigorous study to better quantify peak B (e.g., isolation and identification of peak B and estimation of its relative response factor) is desirable but is outside the scope of this paper. 

The qualitative descriptors in the model experiment were a change of color from yellowish to brown, indicating possible degradation (DoE1), and a change in appearance from transparent to opaque, indicating oversaturation (DoE2). UV-Vis spectroscopy enabled quantification of color of the product by calculating L* values. The system was also shown to be robust and reliable (verification experiments). Only recent developments in spectroscopic equipment made it possible to also include the visible part of the spectra into quantitative color analyses. As the model has shown, the UV–part of the spectra also adds valuable information about the impact of API concentration in the polymer. A follow-on study is aimed to show the potential of UV-Vis for measuring real time API concentration in the polymer melt during HME processes.

In the model, the underlying effects of visual quality attributes became apparent in the absorbance spectra. Changes in color were reflected in a change of absorbance, leading to a shift of the plateau in the visible part of the spectra. It is therefore possible to measure API solubility threshold limit or oversaturation with UV-Vis. Furthermore, Abs at 680 nm was chosen to evaluate this shift and the DoE models identified concentration as the predominant critical parameter and temperature as the second most influential parameter. 

From these analyses, we could immediately conclude that operating close to conditions that are difficult to control increases the risk of unstable results, i.e., temperatures above 150 °C and concentrations higher than 20% *w*/*w*. A decrease in lightness (L*) is the result of a lack of dissolution of API or caused by another effect that scatters light (e.g. bubbles).

UV-Vis might answer the most urgent questions of early phase product development faster and more reliably. With a good risk assessment and a simple set of experiments, it was possible to find, very quickly, in a systematic and multivariate way, that concentration and temperature were most influential parameters that affect color and solubility limit of the API in the polymer during this HME process. 

Moreover, further experiments in direct comparison showed that UV-Vis is far more sensitive than NIR and Raman when it comes to small and very low API concentrations (to be published). The results suggest that UV-Vis spectroscopy could grow out of its niche existence. 

So far, NIR and Raman spectroscopy are the most common in-line PAT systems to use in a QbD-approach. NIR spectroscopy is the most popular technology among the modern process analyzers and has been increasingly used for real-time measurements of critical process parameters and product critical quality attributes, as this technique allows rapid and non-destructive measurements without sample preparations [47,48,49]. However, the complexity of NIR interpretation results from the overlapping of hydrogen absorbance bands of the different functional groups (–CH, –OH, –NH, –SH), requiring chemometrical data for interpretation. A strong calibration and validated model form the basis for a well-predictive NIR measurement model. Raman spectroscopy is used for routine qualitative and quantitative measurements of both inorganic and organic materials. It is popular for raw material identification, as it does not require the use of multivariate modelling [50,51,52,53]. 

The results reported here showed that UV-Vis is a robust and rapid PAT system that can be used to identify the relationship between CPPs and CQAs in HME processes using multivariate approach. Ideally, in a manufacturing situation, the use of UV-Vis spectra as a monitoring tool should be validated first. This case study is more representative of early phase product development, where we had little prior knowledge from the start. We needed to do exploratory work to understand the process conditions and the failure modes first. By definition we could only perform the off-line HPLC, DSC and XRD characterization after we had obtained the samples by continuous manufacture therefore by necessity, the off-line validation of the UV-Vis spectra as an in-line monitoring tool occurred subsequently.

## 5. Conclusions

In-line UV-Vis spectroscopy was used as a fast-working PAT tool for early phase product development during HME. A systematic approach based on sequential design of experiments was used to screen, optimize and verify process conditions using multivariate statistical analysis to quantify the relationship between critical process parameters (CPPs) and critical quality attributes (CQAs). Real time data of L* and absorbance on the visible region were used to identify solubility threshold limit or oversaturation of API in the polymer matrix. The optimum ranges for temperature (140 °C), API concentration (20% *w*/*w*), screw speed (200 rpm) and solid feed rate (6 g/min) were determined and a potential design space was proposed for the acceptable process conditions. Temperature and concentration were found to have strong interaction effects.

Piroxicam was found to be miscible in Kollidon^®^ VA64 up to 20% *w*/*w* drug loading. Results from off-line characterization using DSC, XRD and HPLC supported the in-line UV-Vis results. 

In-line UV-Vis spectroscopy might become a useful system for other extrusion applications. API solubility can be visualized and monitored by measuring absorbance and calculating L*, a*, b* color values [19,34]. Moreover, many API show a pronounced absorbance in the UV spectrum and/or in the visible part of the spectrum, making in-line UV-Vis spectroscopy a potentially widespread system for monitoring quality in pharmaceutical HME processes.

A follow-on study is aimed to show the potential of UV-Vis for measuring API concentration in the polymer melt and API content uniformity in powder mixtures (HME upstream and downstream processes).

## Figures and Tables

**Table 1:** DoE1 Screening study with appearance pass/fail outcome.

Run Number	API Concentration % *w/w*	Die Temperature °C	Screw Speed rpm	Solid Feed Rate g/min	L* %	Abs at 680 nm	Extrudate Appearance	Extrudate Colour	Extrudate Pass/Fail
1	15	150	100	4	96.62	0.01	transparent	yellow	pass
2	15	150	300	8	60.18	0.43	opaque	orange	fail
3	15	170	100	8	48.91	0.50	opaque	orange	fail
4	15	170	300	4	65.14	0.69	opaque	dark red	fail
5	35	150	300	4	10.07	0.86	opaque	orange	fail
6	35	150	100	8	49.46	1.86	opaque	very dark red	fail
7	35	170	300	8	53.59	0.55	opaque	orange	fail
8	35	170	100	4	40.02	0.49	opaque	red	fail
9	25	160	200	6	54.23	0.57	opaque	dark orange	fail
10	25	160	200	6	55.38	0.55	opaque	dark orange	fail

**Table 2:** Analysis of variance for response L* (DoE1).

**Source**	**DF**	**Sum of Squares**	**Mean Square**	**F ratio**			
Model	6	4138.7755	689.796	210.8323			
Error	3	9.8153	3.272	**Prob > F**			
C. Total	9	4148.5908		0.0005*			
**Summary of Fit**							
RSquare		0.997634					
RSquare Adj		0.992902					
Root Mean Square Error		1.808805					
Mean of Response		53.36					
Observations		10					
**Sorted Parameter Estimates**							
**Term**			**Estimate**	**Std Error**	**t Ratio**		**Prob > |t |**
Concentration (% *w*/*w*) (15,35)			−14.71375	0.639509	−23.01	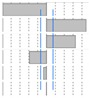	0.0002*
Concentration (% *w*/*w*) * Solid feed rate (g/min)			13.20375	0.639509	20.65		0.0002*
Concentration (% *w*/*w*) * Temperature (°C)			9.60375	0.639509	15.02		0.0006*
Screw speed (rpm) (100,300)			−5.75375	0.639509	−9.00		0.0029*
Temperature (°C) (150,170)			−1.08375	0.639509	−1.69		0.1887
Solid feed rate (g/min) (4,8)			0.03625	0.639509	0.06		0.9584

**Table 3:** Analysis of variance for response Abs at 680 nm (DoE1).

**Source**	**DF**	**Sum of Squares**	**Mean Square**	**F ratio**			
Model	8	2.0420900	0.255261	1276.306			
Error	1	0.0002000	0.000200	**Prob > F**			
C. Total	9	2.0422900		0.0216*			
**Summary of Fit**							
RSquare		0.999902					
RSquare Adj		0.999119					
Root Mean Square Error		0.014142					
Mean of Response		0.651					
Observations		10					
**Sorted Parameter Estimates**							
**Term**			**Estimate**	**Std Error**	**t Ratio**		**Prob > |t |**
Concentration (% *w*/*w*) * Temperature (°C)			−0.30375	0.005	−60.75	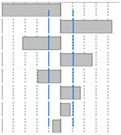	0.0105*
Concentration (% *w*/*w*) (15,35)			0.26625	0.005	53.25		0.0120*
Concentration (% *w*/*w*) * Screw speed (rpm)			−0.19375	0.005	−38.75		0.0164*
Solid feed rate (g/min) (4,8)			0.16125	0.005	32.25		0.0197*
Temperature (°C) (150,170)			−0.11625	0.005	−23.25		0.0274*
Concentration (% *w*/*w*) * Solid feed rate (g/min)			0.10375	0.005	20.75		0.0307*
Concentration (% *w*/*w*) * Concentration (% *w*/*w*)			0.11375	0.01118	10.17		0.0624
Screw speed (rpm) (100,300)			−0.04125	0.005	−8.25		0.0768

**Table 4:** Parameters and responses for DoE2, central composite design.

Run Number	API Concentration % *w/w*	Die Temperature °C	Screw Speed rpm	Solid Feed Rate g/min	L* %	Abs at 680 nm	Extrudate Appearance	Extrudate Colour	Extrudate Pass/Fail
1	20	130	200	6	94.58	0.034	transparent	uniform yellow	pass
2	20	140	200	6	94.69	0.035	transparent	uniform yellow	pass
3	20	150	200	6	94.11	0.039	transparent	uniform yellow	pass
4	25	130	200	6	68.05	0.395	opaque	yellowish	fail
5	25	140	200	6	87.08	0.126	opaque	yellowish	fail
6	25	140	200	6	87.44	0.133	opaque	yellowish	fail
7	25	140	200	6	87.51	0.131	opaque	yellowish	fail
8	25	150	200	6	92.62	0.054	opaque	yellowish	fail
9	30	130	200	6	39.77	0.930	opaque	yellowish	fail
10	30	140	200	6	55.18	0.612	opaque	yellowish	fail
11	30	150	200	6	78.29	0.239	opaque	yellowish	fail

**Table 5:** Analysis of variance for response L* (DoE2).

**Source**	**DF**	**Sum of Squares**	**Mean Square**	**F ratio**			
Model	4	3249.5224	812.381	61.5001			
Error	6	79.2566	13.209	**Prob > F**			
C. Total	10	3328.7790		<.0001*			
**Summary of Fit**							
RSquare		0.97619					
RSquare Adj		0.960317					
Root Mean Square Error		3.634478					
Mean of Response		79.93818					
Observations		11					
**Sorted Parameter Estimates**							
**Term**			**Estimate**	**Std Error**	**t Ratio**		**Prob> |t |**
Concentration (% *w*/*w*) (20,30)			−18.35667	1.483769	−12.37	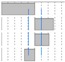	<.0001*
Temperature (°C) (130,150)			10.436667	1.483769	7.03		0.0004*
Concentration (% *w*/*w*) * Temperature (°C)			9.7475	1.817239	5.36		0.0017*
Concentration (% *w*/*w*) * Concentration (% *w*/*w*)			−8.436667	2.200786	−3.83		0.0086*

**Table 6:** Analysis of variance for response Abs at 680 nm (DoE2).

**Source**	**DF**	**Sum of Squares**	**Mean Square**	**F ratio**			
Model	4	0.82234070	0.205585	109.6522			
error	6	0.01124930	0.001875	**Prob > F**			
C. Total	10	0.83359000		<.0001*			
**Summary of Fit**							
RSquare		0.986505					
RSquare Adj		0.977508					
Root Mean Square Error		0.0433					
Mean of Response		0.248					
Observations		11					
**Sorted Parameter Estimates**							
**Term**			**Estimate**	**Std Error**	**t Ratio**		**Prob > |t |**
Concentration (% *w*/*w*) (20,30)			0.2788333	0.017677	15.77	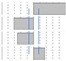	<.0001*
Temperature (°C) (130,150)			−0.171167	0.017677	−9.68		<.0001*
Concentration (% *w*/*w*) * Temperature (°C)			−0.174	0.02165	−8.04		0.0002*
Concentration (% *w*/*w*) * Concentration (% *w*/*w*)			0.1470333	0.026219	5.61		0.0014*

**Table 7:** Process Conditions Verification study.

Run Number	API Concentration % *w/w*	Die Temperature °C	Screw Speed rpm	Solid Feed Rate g/min	L* %	Abs at 680 nm	Extrudate Appearance	Extrudate Colour	Extrudate Pass/Fail	Operator
DoE2 Run2	20	140	200	6	94.69	0.035	transparent	light yellow	pass	A
Verification run 1	20	140	200	6	98.58	-0.006	transparent	light yellow	pass	A
Verification run 2	20	135	200	6	95.30	0.016	transparent	light yellow	pass	B
Verification run 3	20	140	300	6	93.90	0.019	transparent	light yellow	pass	B
Verification run 4	20	135	200	6	95.20	0.015	transparent	light yellow	pass	B

**Table 8:** Calculation of sample concentrations based on average peak areas at 330 and 360 nm for DoE1 = Screening Design, DoE2 = optimization and V = verification.

DoE	Sample Label	Run Sample Concentration (% *w/w*)	HPLC Sample Concentration (mg/mL)	Peak A Average Area (mAU*s) at 360 nm	Peak B Average Area (mAU*s) at 330 nm	Relative Area Peak A (%)	Relative Area Peak B (%)	Measured Concentration (mg/mL)
DoE1	Run 1	15	0.0374	1822.12	16.5503	98.93	1.07	0.0319
	Run 2	15	0.0372	1838.15	21.3744	98.66	1.34	0.0322
	Run 3	15	0.0374	1714.94	31.1404	97.90	2.10	0.0300
	Run 4	15	0.0374	1440.43	52.8780	95.87	4.13	0.0250
	Run 5	35	0.0875	3076.03	32.0652	98.81	1.19	0.0546
	Run 6	35	0.0870	4011.21	32.2373	99.09	0.91	0.0716
	Run 7	35	0.0868	3546.24	22.8981	99.24	0.76	0.0632
	Run 8	35	0.0873	4330.38	48.7399	98.57	1.32	0.0773
	Run 9	25	0.0621	2889.15	29.3632	98.82	1.18	0.0513
	Run 10	25	0.0623	2897.23	29.6303	98.84	1.16	0.0514
DoE2	Run 2	20	0.0498	2551.42	18.4762	99.25	0.75	0.0451
	Run 6	25	0.0625	3484.15	20.3990	98.23	1.77	0.0620
	Run 10	30	0.0743	3963.65	21.7395	99.40	0.60	0.0707
Verification	Run 1	20	0.0486	2539.20	6.6582	99.70	0.30	0.0449

**Table 9:** Analysis of variance for response relative to peak area B (%).

**Summary of Fit**							
RSquare			0.955				
RSquare Adj			0.94375				
Root Mean Square Error			0.218715				
Mean of Response			1.262383				
Observations (or Sum Wgts)			41				
**Analysis of Variance**							
**Source**	**DF**	**Sum of Squares**	**Mean square**	**F ratio**			
Model	8	32.485939	4.06074	84.8882			
Error	32	1.530764	0.04784	**Prob > F**			
C. Total	40	34.016703		<.0001*			
**Sorted Parameter Estimates**							
**Term**			**Estimate**	**Std Error**	**t Ratio**	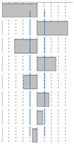	**Prob> |t |**
Die Temperature (°C)			0.0468694	0.002979	15.73		<.0001*
Concentration (%*w*/*w*) * Die Temperature (°C)			−0.00447	0.000389	−11.48		<.0001*
Concentration (% *w*/*w*)			−0.03328	0.004532	−7.34		<.0001*
Die Temperature (°C) * Solid feed rate (g/min)			−0.016132	0.002232	−7.23		<.0001*
Screw Speed (rpm)			0.0019133	0.000507	3.78		0.0007*
Solid Feed Rate (g/min)			−0.075049	0.025333	−2.96		0.0057*
0.Die Temperature (°C) * Screw Speed (rpm)			0.0001133	4.465 × 10^−5^	2.54		0.0162*
Die Temperature (°C) Die Temperature (°C)			0.0006288	0.000347	1.81		0.0796
